# The effect of augmented reality storybooks on the story comprehension and retelling of preschool children

**DOI:** 10.3389/fpsyg.2024.1459264

**Published:** 2024-10-08

**Authors:** Emine Ela Şimşek

**Affiliations:** Department of Preschool Education, Faculty of Education, Akdeniz University, Antalya, Türkiye

**Keywords:** augmented reality, story-retelling, story comprehension, preschool children, retelling

## Abstract

This study aimed to compare the retelling and story comprehension performance of two groups of preschool children—an experimental and a control group—who experienced printed and augmented reality storybooks. The participant group consisted of 90 participants, with 45 in the experimental group (22 girls, 23 boys) and 45 in the control group (21 girls, 24 boys). The average age of the children was 54.2 months. In the study, the researcher evaluated children’s story-retelling performance using the rubric and used a Story Comprehension Test to measure their story comprehension performance. The researchers utilized the ROAR application to incorporate augmented reality content into the books. In the pre-test phase, the teachers read the designated texts to the children in the experimental and control groups. After the eight-week break, the control group experienced the same texts with printed books, while the experimental group experienced them with augmented reality support. Each child participated individually in the reading process with the teacher. In the study, teachers asked the children to retell the story and asked the questions from the Story Comprehension Test at the end of each book reading section for pre-test and post-test measurements. The pre-test results revealed no significant difference in the two groups’ story-retelling and Story Comprehension Test scores. The post-test results indicated a significant difference in the story-retelling performance and Story Comprehension Test scores between the experimental and control groups, favoring the experimental group. Based on these findings, the augmented reality content can potentially enhance children’s retelling and story comprehension performances.

## 1 Introduction

The preschool period is a critical phase during which the foundations of children’s cognitive, emotional, social, and language development are established. The educational materials and activities provided to children during this period significantly influenced their future learning and life skills. Among these materials, picture storybooks hold a special place. Reading picture storybooks can contribute to children’s acquisition of knowledge, learning to communicate, forming their cultural identities, enhancing their imagination, and fulfilling their desire for discovery (Carrión Candel et al., 2020; Takacs and Bus, 2018; Yamaji et al., 2020). Additionally, it is known that picture storybooks are beneficial for developing children’s current literacy skills (Fears and Lockman, 2020; Hu and Commeyras, 2008; Wang, 2022) and language skills (Grolig et al., 2019). Story reading provides a meaningful context for children to acquire skills such as listening comprehension, retelling, following plot structures, and inferential thinking (Collins, 2016; Florit et al., 2011; Morrow, 1985; Strasser and Río, 2014). By contributing to language development, reading stories enables children to learn new words and grasp story elements (Breit-Smith et al., 2017; Han and Neuharth-Pritchett, 2015). Furthermore, it has been determined that reading storybooks contributes to language skills and academic achievement from the preschool period to university (Mol and Bus, 2011). Therefore, it is worth considering how children’s interaction with picture storybooks can be supported.

With technological advancements, storybooks have started to be presented in different formats. As technology-enhanced books become prevalent, they have gained increased attention from researchers. Indeed, numerous studies have been conducted on topics such as electronic books (Lauricella et al., 2014), interactive books (Lim et al., 2021; Zipke, 2017), and multimedia book applications (Zhou and Yadav, 2017; Tsou et al., 2006). These books, used today, have also transformed children’s early literacy experiences compared to previous generations. Children nowadays have the opportunity to experience both printed and digital books (Kucirkova, 2016). Each type of book has its distinctive features. Printed storybooks typically consist of static illustrations alongside the text, whereas digital storybooks offer multimedia features that enhance the story’s content and allow children to listen to the narrative (Merjovaara et al., 2020; O'Byrne et al., 2018). Augmented reality technology, which has become increasingly prevalent in recent years, combines printed and digital books’ features, enabling innovative storybooks to be created (Danaei et al., 2020; Şimşek and Direkci, 2023).

According to Azuma (1997), augmented reality is the innovative integration of real-world and virtual elements, enabling seamless interaction between the two realms. This technology presents a format where natural environment images are used as a background, and digital content (such as animation, 3D content, etc.) is overlaid on top, enabling user interaction (Billinghurst et al., 2001). In this regard, it bridges the real and virtual worlds through computer screens or mobile applications. The relevant literature has listed numerous benefits of augmented reality applications. These studies have shown that augmented reality applications reduce cognitive load (Alvarez-Marin and Velazquez-Iturbide, 2021; Cheng, 2015; Keller et al., 2021; Ibili and Billinghurst, 2019; Wilson, 2015; Wen, 2021), facilitates comprehension of abstract concepts (Yoon et al., 2017), enhance learning performance (Chang et al., 2015; Chen, 2019; Chin et al., 2021; Ferrer-Torregrosa et al., 2015), increase learning motivation (Amores-Valencia et al., 2022; Chiang et al., 2014; Di Serio et al., 2013; Ferrer-Torregrosa et al., 2015; Khan et al., 2019), and support academic achievement (Barreira et al., 2012; Duenser, 2008; Ibáñez et al., 2020; Ömurtak and Zeybek, 2022). Furthermore, augmented reality applications are enjoyable (Wojciechowski and Cellary, 2013) and engaging (Delello, 2014; Fidan and Tuncel, 2019; Perez-Lopez and Contero, 2013; Tomi and Rambli, 2013), as well as contributing to the development of positive attitudes toward the subject among students (Şahin and Yılmaz, 2020; Akçayır et al., 2016).

One of the applications of augmented reality technology is in storybooks. Augmented reality is often used in storybooks to enhance immersion through animations and virtual pop-ups (Kljun et al., 2019). Augmented reality storybooks incorporate characters and objects in 2D or 3D animations, accompanied by diverse technical effects such as videos, sounds, and interactive functions like touch, zoom, and rotate (Alhumaidan et al., 2018). This gives readers a reading experience that dynamically interacts with virtual content in a real-world setting (Cheng and Tsai, 2014; Green et al., 2019). According to Santos et al. (2016), when children engage with augmented reality storybooks, they can visualize information within a context-rich environment. This immersive experience enables them to forge genuine connections between educational content and the real world. Previous research has reported a range of advantages of augmented reality storybooks, including their ability to be engaging (Soon et al., 2022), enhance attention (Aydoğdu, 2022), contribute to learning (Duenser et al., 2012), improve empathy skills (Gil et al., 2014), enable interactive storytelling (Zhou et al., 2004), increase reading motivation (Roumba and Nicolaidou, 2022), and foster reading habits (Anuardi et al., 2022). One of the benefits mentioned in the literature is that the multisensory stimuli provided by augmented reality contribute to understanding the story (Ertem, 2009; Kao et al., 2016; Pan et al., 2021).

When reviewing the relevant literature, studies have been conducted on the effects of augmented reality storybooks on comprehension at different educational levels. Duenser (2008) has found through these studies that augmented reality storybooks offer a favorable learning environment that effectively supports readers with lower abilities. Moreover, in many studies, it has been found that students who read augmented reality storybooks have higher comprehension performance compared to groups reading printed books (Çetinkaya Özdemir and Akyol, 2021; Ebadi and Ashrafabadi, 2022; Şimşek and Direkci, 2023). Additionally, students who experienced augmented reality storybooks demonstrated better performance in retelling (Danaei et al., 2020), answering inferential questions (Liu et al., 2024), and learning retention (Bursalı and Yılmaz, 2019). However, Şimşek and Direkci's (2023) study found no significant difference in basic comprehension levels between augmented reality intervention and traditional reading. Similarly, Tobar-Munoz et al. (2017) did not detect a significant difference in total scores between groups in their study. Overall, when examining the studies in the relevant literature, it can be said that augmented reality storybooks have the potential to support comprehension.

In the conducted studies, comprehension processes have generally been evaluated through tests (Bursalı and Yılmaz, 2019; Çetinkaya Özdemir and Akyol, 2021; Ebadi and Ashrafabadi, 2022; Şimşek and Direkci, 2023; Tobar-Munoz et al., 2017) and retelling (Çetin and Ulusoy, 2023). Limited studies combine retelling and testing (Danaei et al., 2020; Liu et al., 2024). Therefore, this study used both assessment methods to yield more in-depth results. Furthermore, when examining studies conducted on augmented reality and language instruction in the literature, it can be observed that there is a scarcity of augmented reality studies with preschool children (Cai et al., 2022; Parmaxi and Demetriou, 2020). There is evidence of a connection between preschool children’s listening comprehension skills and elementary school children’s reading comprehension skills (Kendeou et al., 2007; Kendeou et al., 2009). Indeed, several studies have found that understanding narratives in preschool years predicts later reading comprehension (Kim, 2016; Language and Reading Research Consortium and Chiu, 2018). Therefore, the impact of augmented reality storybooks, which have clear findings supporting reading comprehension in elementary and later grades, on preschool children is an essential research topic.

### 1.1 Current study

This study examined and compared the retelling and story comprehension performance of preschool children who engage with printed and augmented reality storybooks. The following research questions were addressed in the study:

RQ1: What was the difference in retelling performance between children participating in the traditional storybook narration activity and those participating in the augmented reality storybook narration activity?

RQ2: What was the difference in story comprehension performance between children participating in the traditional storybook narration activity and those participating in the augmented reality storybook narration activity?

## 2 Methodology

### 2.1 Participants

This study was conducted in preschools located in Antalya, Türkiye. The participant group consisted of 90 participants, with 45 in the experimental group (22 girls, 23 boys) and 45 in the control group (21 girls, 24 boys). The average age of the children was 54.2 months. The children were randomly assigned to either the experimental group, where they experienced augmented reality storybooks under the teacher’s guidance, or the control group, where the teacher individually read to each participant. The participants were enrolled in four classrooms, and the teachers were included in the research process to facilitate the children’s comfortable expression. This choice was made to ensure that the children could express themselves freely. Moreover, preliminary interviews indicated that teachers and children were accustomed to using technological applications in the classroom.

### 2.2 The augmented reality storybook and application

This research aimed to provide children with an engaging reading experience by transforming three selected picture books into an augmented reality-supported storybook format. In line with this objective, the opinions of the teachers in the classrooms where the application was implemented were obtained to determine the suitability of the books titled “Aç Tırtıl (The Very Hungry Caterpillar),” “Kafası Karışık Bukalemun (The Mixed-Up Chameleon)” and “Huysuz Uğurböceği (The Grouchy Ladybug)” for children and the research. After identifying the books, a digital content scan was conducted to convert the books into an augmented reality-supported storybook format. Various video contents were identified, and necessary permissions were obtained to adjust the videos. The augmented reality content to be integrated into the reading text should contribute to understanding and consistency with the text. Thus, both the texts and visuals had been prepared in a manner consistent with the narrative flow. Consequently, each page in the printed book aligned with the pages of the augmented reality storybook. The differences were achieved by adding animations and voiceovers on certain pages. To highlight these pages, a small tablet icon had been placed on them. As a result, when teachers read the book with children, the children also heard the same sentences that were voiced using the augmented reality content. In this study, the ROAR application had been selected as the augmented reality tool. The ROAR application allows for content creation via the website “https://theroar.io/.” Through this website, the visuals in the book had been matched with the digital content developed by the researcher. The ROAR application was chosen due to its utility for integrating digital content into printed text and its ability to facilitate the process for teachers during implementation. After the content matching was completed, the associated digital content was triggered when the ROAR application was opened on a tablet and pointed at the visual in the printed book. Consequently, the augmented reality content was activated solely on the designated visual. Data Collection Tools.

In the study, the researcher evaluated children’s story-retelling performance using the rubric suggested by Cruz de Quiros et al. (2012). This rubric included indicators such as “Setting When and Where, Characters, Event/Plot, Problem, and Solution.” Each indicator was assessed on a scale of 3 points, resulting in a score range of 0–15 for each story. This rubric was selected to evaluate children’s storytelling performance more systematically in this study. In addition to assessing children’s retelling performance, a Story Comprehension Test was used to measure their story comprehension performance. This test consists of nine open-ended questions prepared based on the three picture books. The researcher developed the questions. A draft scoring rubric was prepared to ensure a reliable evaluation of the open-ended questions. The draft rubric scoring key was presented for evaluation by the four teachers participating in the study. As a result of the assessment, the questions prepared by the researcher, specifically the 3rd question (“What happened after the caterpillar became enormous?”) and the 4th question (“What would happen when the chameleon got cold?”), were revised as follows. Thus, a consensus was reached, and the final version of the rubric scoring key was established. According to the final scoring rubric, incorrect answers in the open-ended questions were evaluated as 0 points, partially correct answers as 1 point, and correct answers as 2 points. Additionally, two teachers participated in the evaluation process alongside the researcher, and the agreement between the results was assessed. Here are some sample questions from the Story Comprehension Test:

1. *Name four fruits that the caterpillar ate.*
2. *How did the caterpillar’s stomachache go away?*
3. *What happened after the caterpillar became enormous and fat?*
4. *What would happen when the chameleon got cold and hungry?*
5. *What would the chameleon do when it was hungry?*
6. *Name four animals that the chameleon wanted to resemble.*
7. *Name four animals that the grumpy ladybug encountered.*
8. *What did the wet, hungry, and tiredladybug eat for dinner? Who did it eat with?*
9. *What were the fireflies doing when the ladybugs fell asleep?*

### 2.3 Procedures

**Figure 1:**
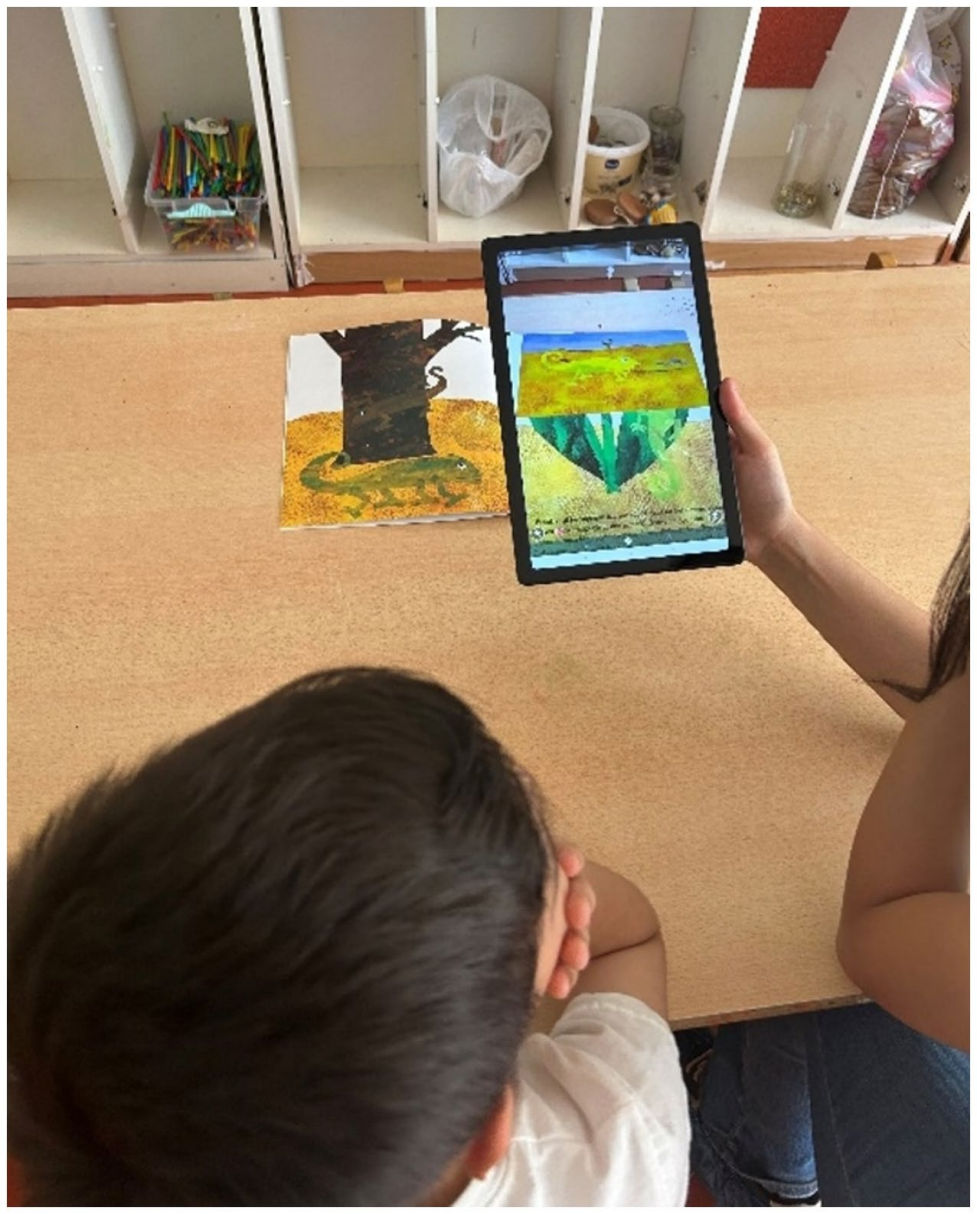
Augmented reality storybook application view.

This study is a pre-test, post-test, and control group design conducted as a quasi-experimental study. Before the implementation, ethical approval was obtained from the Ethics Committee of Akdeniz University, and permission to conduct the study was obtained from the Antalya Provincial Directorate of National Education. Subsequently, meetings were held with the teachers working in the implementation schools to provide information about the study. The implementation phase began once the teachers fully understood the principles of the augmented reality storybook. The researcher presented in the classroom to provide technical support and oversee the implementation process. In the initial phase of the study, teachers read three printed texts to children in both the experimental and control groups. After each book was read, the children were asked to retell the story. Subsequently, questions from the Story Comprehension Test were posed to the children. The teacher recorded the children’s storytelling performances and their responses to the comprehension test during both the pre-test and post-test. These processes were conducted in the classroom environment where the books were read. The pre-test phase was concluded after the children answered the questions, and the teacher recorded responses. An eight-week break was provided with the assumption that the content of the books could be remembered. During this period, the teachers did not intervene in the classrooms regarding this research. However, 1 week before the post-test phase with the experimental group, the teachers read the “Minik Tohum (The Tiny Seed)” book, which was not included in the assessment process, together with the children as an augmented reality-enriched reading experience. This practice was conducted to familiarize the children with augmented reality storybooks and make them feel more comfortable during the reading process in the post-test application. Tablet computers were used during the implementation process, and the researcher brought these devices to the classroom with the necessary applications installed. After an 8-week interval, the teacher read the same printed books to the control group. Meanwhile, the experimental group experienced the augmented reality storybooks. During this process, the teacher read the printed book to the children and used the tablet on the visuals that required activation of the augmented reality content. The augmented reality application activated an animation on the visual, which was accompanied by a narration of the story. As a result, the teacher remained more passive in situations where the augmented reality application was used. Visuals related to this application are presented in Figure 1. Each child participated individually in the reading process with the teacher. After the reading process, each child retold the story and answered the questions from the Story Comprehension Test. The children were informed that there were no right or wrong answers to the questions and that it was not an exam to encourage them to think freely. After collecting the post-test data, the analysis phase was initiated.

### 2.4 Data analysis

Normality tests were initially performed to determine the analysis to be conducted in the study. Due to the normal distribution of the data, an independent samples *t*-test was used to examine possible differences between the experimental and control groups in the analysis of pre-test results. Similarly, a paired samples *t*-test was conducted to determine within-group differences in the analysis of post-test results, considering the normal distribution of the data. Furthermore, independent samples *t*-tests were utilized to examine potential variances between the experimental and control groups. Cohen’s *d* was employed to determine the effect size when a statistically significant difference was found. Kendall’s W Concordance coefficient was calculated to evaluate the agreement between the two teachers and the researcher involved in the Story Comprehension Test evaluation process. The results indicated a concordance coefficient of 0.964 for the pre-test and 0.973 for the post-test.

## 3 Results

The study’s data analysis commenced with a normality test. It was determined that the retelling and Story Comprehension Test scores for both the experimental and control groups followed a normal distribution. Subsequently, the analysis of the data obtained from the pre-tests revealed no significant difference in the retelling and Story Comprehension Test scores between the children in the two groups within a 95% confidence interval. The means, standard deviations, and results of the independent samples *t*-test for the pre-test processes are presented in Table 1.

**Table 1:** Mean, standard deviation and results of the independent samples *t*-test for the pre-test.

	Group	*N*	Mean	SD	*t*	*p*
Retelling	Experimental group	45	23.533	5.020	1.340	0.184
	Control group	45	22.155	4.728		
Story comprehension test	Experimental group	45	11.755	2.101	1.392	0.167
	Control group	45	11.177	1.825		

Upon reviewing Table 1, it is evident that there was no notable difference in the story-retelling performance in the experimental and control groups (*t* = 1.340, *p* > 0.05). Similar results were obtained for the Story Comprehension Test scores between the groups (*t* = 1.392, *p* > 0.05). Therefore, it can be concluded that there is no significant difference in the comprehension and retelling performance of the children who participated in the study without any intervention. No intervention was conducted for the control group during the post-test phase of the study. However, it was found that the retelling score of the control group increased from 22.115 to 23.622. Nevertheless, no significant distinction was observed between the pre-test and post-test scores of the control group (*t* = −1.843, *p* > 0.05). Similarly, the Story Comprehension Test scores of the control group increased from 11.177 to 11.888. However, there was no significant difference between the scores (*t* = −1.907, *p* > 0.05). In contrast, significant differences were discovered in the pre-test and post-test scores of the experimental group, indicating notable variations in retelling performance (*t* = −13.984, *p* < 0.05) and Story Comprehension Test (*t* = −10.715, *p* < 0.05). Furthermore, the disparity between the post-test scores of the experimental and control groups was assessed. The means, standard deviations, and results of the independent samples *t*-test for the post-test are presented in Table 2.

**Table 2:** Mean, standard deviation and results of the independent samples *t*-test for the post-test.

	Group	*N*	Mean	SD	*t*	Cohen *d*
Retelling	Experimental group	45	28.822	3.978	5.701*	1.202
	Control group	45	23.622	4.648		
Story comprehension test	Experimental group	45	14.555	2.388	6.075*	1.281
	Control group	45	11.888	1.721		

*p < 0.05.

Based on the findings in Table 2, a significant difference was evident between the retelling performance of the story in the experimental and control groups (*t* = 5.701, *p* < 0.05). Similar results were found for the Story Comprehension Test scores indicating a significant difference between the two groups (*t* = 6.075, *p* < 0.05). The findings demonstrated that the augmented reality intervention better supported the understanding of the story compared to traditional narration. Additionally, the retelling performance of the children also improved with the augmented reality intervention. The effect size analyses indicated a substantial effect size for both the retelling and Story Comprehension Test scores, highlighting the substantial difference between the two groups (Cohen, 1988).

Given the information provided, the research questions can be addressed and answered in the following manner:

RQ1: The retelling performance of children who participated in the augmented reality storybook narration activity is better than that of children who participated in the traditional story narration activity.

RQ2: The story comprehension performance of children who participated in the augmented reality storybook narration activity is better than that of children who participated in the traditional story narration activity.

## 4 Discussion

This study delved into the differences in comprehension levels between the control group, which was exposed to printed books with traditional storytelling, and the experimental group, which engaged in augmented reality storybook narration. The children were tasked with retelling the stories they heard and answering the questions in the Story Comprehension Test to gauge this difference. In the pre-test phase, there was no discernible difference between the two groups regarding retelling and Story Comprehension Test scores. However, the final test results unveiled a compelling revelation-augmented reality storybook narration that significantly bolstered comprehension and retelling, surpassing the effectiveness of traditional storytelling using printed books.

According to the research findings, children who participated in augmented reality storybook narration performed better in retelling the story than in traditional storytelling activities. Augmented reality studies conducted with preschool children demonstrate that this technology is well-liked by children (Rambli et al., 2013), provides an enjoyable learning environment for children (Albayrak and Yılmaz, 2022), enhances children’s motivation (Aydoğdu, 2022), and captures children’s interest (Bülbül and Özdinç, 2022). Therefore, augmented reality storybooks may have also captured children’s interest and contributed to their comprehension. It can be argued that this situation supports children’s performance in retelling the story. Another factor that may support children’s performance is the multimedia features within the augmented reality storybooks. Elements such as the tone of voice of narrators that can convey emotions and moods, music, and sound effects may capture the readers’ attention and immerse them in the story, potentially enhancing their imagination as if they were the narrative’s main characters. Additionally, animations can better depict characters’ facial expressions and gestures than static images (Danaei et al., 2020). These features provide augmented reality with a rich and vibrant reading experience (ChanLin, 2018). Furthermore, augmented reality support enhances students’ learning experiences by providing interactivity and visual representation (Bujak et al., 2013; Teng et al., 2018). Consequently, with their existing features, augmented reality storybooks can support children’s storytelling performance. Indeed, previous studies in the literature also support these results. In their study, Liu et al. (2024) compared comprehension levels between two groups. One group read an augmented reality storybook, while the other read a printed picture book. The results showed that participants were particularly successful in the narrative structures related to the setting and plot during the retelling process.

Furthermore, using augmented reality, storybooks helped participants better understand the characters and their emotional changes (Liu et al., 2024). In contrast to this study, Danaei et al. (2020) found no difference in the setting between groups in their research. However, when looking at the total scores, it was determined that augmented reality storybooks improved retelling performance (Danaei et al., 2020). Another research conducted by Çetin and Ulusoy (2023) with third-grade students also supports these results. Upon reviewing the literature, it can be observed that the participants in the relevant studies were elementary and post-elementary school students. Therefore, the results regarding how augmented reality storybooks affect the retelling performance of preschool children are essential for the relevant literature.

According to the research results, children who participated in augmented reality storybook narration activities exhibited better performance in story comprehension than in traditional storytelling activities. According to the study by Takacs and Bus (2016), children exposed to an animated book understand the story better than those who view static images. In this regard, the ability of augmented reality to integrate verbal and non-verbal information and provide dynamic interaction may assist children in comprehending the text (Alsowat, 2016; Dunleavy et al., 2009). Additionally, augmented reality storybooks can offer children a sense of presence and an immersive environment through audiovisual content (Ebadi and Ashrafabadi, 2022). Consequently, it can be concluded that augmented reality storybooks support children’s story comprehension performance. Studies in the literature indicate that augmented reality storybooks enhance users’ comprehension performance. Yılmaz et al. (2017) determined that children who interacted with these books displayed strong story comprehension in their study with five- and six-year-olds. The majority of other studies conducted on this topic involve students in elementary and higher grades, and these studies also provide evidence that augmented reality storybooks support comprehension (Bursalı and Yılmaz, 2019; Çetinkaya Özdemir and Akyol, 2021; Ebadi and Ashrafabadi, 2022). Some of these studies show that augmented reality interventions do not create a significant difference compared to printed books in simple comprehension-based questions (Danaei et al., 2020; Şimşek and Direkci, 2023). However, users perform better in answering implicit questions (Danaei et al., 2020; Liu et al., 2024; Şimşek and Direkci, 2023) and higher-level questions related to reorganization, evaluation, and appreciation compared to printed books (Şimşek and Direkci, 2023). Augmented reality storybooks can support users’ comprehension performance with their ability to appeal to different senses and make the story more tangible. For example, children who interacted with an augmented reality storybook were more likely to provide correct answers to the question, “What were the fireflies doing when the ladybugs fell asleep?” The reason behind this could be that the visuals supported by sound and video make it easier to follow the flow of the story.

This study, along with various studies in the literature, provides evidence that augmented reality storybooks support users’ comprehension performance. Enriching the learning experience using multimedia content, such as text-to-speech, video, and interactive elements, can be a reason for this observed difference (Billinghurst and Duenser, 2012; Yuen et al., 2011). Indeed, incorporating information through multiple channels, encompassing both auditory and visual modalities, has contributed to more effective learning and better retention than processing information through a single channel (Bus et al., 2015). Thus, multimedia content can reduce cognitive load and enhance the story’s comprehensibility (Kao et al., 2016). The relevant literature also supports the idea that multimedia content can enhance preschool children’s comprehension (Altun, 2024) and retelling performance (Crawshaw et al., 2020; Diehm et al., 2020). However, the integrated multimedia content must be consistent with the text. Multimedia content that is not aligned with the story may distract children’s attention from the narrative and increase cognitive load, thereby reducing children’s comprehension performance (Chang et al., 2011; Takacs and Bus, 2016; Takacs et al., 2015).

## 5 Conclusion, limitations and recommendations

This study compared the comprehension levels of preschool children who experienced printed and augmented reality storybooks. In this context, children’s books titled “Aç Tırtıl (The Very Hungry Caterpillar),” “Kafası Karışık Bukalemun (The Mixed-Up Chameleon)” and “Huysuz Uğurböceği (The Grouchy Ladybug)” were enriched with augmented reality content. Subsequently, teachers read the books in printed and augmented reality-supported formats and engage in activities. In order to compare the groups, children retold the stories and answered the questions from the Story Comprehension Test. The results indicated that children who listened to augmented reality storybooks demonstrated higher comprehension and retelling performance than children who listened to printed storybooks. The consistently high scores in both the test and retelling provide in-depth evidence supporting the effectiveness of augmented reality storybooks in enhancing comprehension. Additionally, the limited data available in the literature regarding preschool children makes the findings of this study noteworthy.

This study has several limitations. Firstly, the participants were children between the ages of four and five. Thus, the results are limited to this age range. However, future studies could involve children in different age groups within the preschool period, enabling broader interpretations regarding the impact of augmented reality storybooks on preschool children’s comprehension and retelling performance.

Additionally, the data in this study is limited to 90 children. While studies testing new technological devices and applications generally have smaller sample sizes, a more significant number of studies are needed for the generalizability of the data. The current study’s lack of a follow-up assessment limits the research. Therefore, there is an urgent need for future studies should include follow-up assessments that could provide insights into the lasting effects of augmented reality interventions. The researcher did not use multimedia content on every page in the augmented reality storybooks created in this study. This decision was made to maintain the narrative of a storybook and avoid creating an environment solely focused on watching videos. The integration of multimedia content into the books was done in consultation with teachers and placed in locations deemed appropriate by the researcher. As there are currently no established design principles for augmented reality storybooks regarding factors such as appropriate duration and frequency of multimedia experiences in the literature, future studies should focus on developing design principles that cater to different age groups and align with the characteristics of different texts. Furthermore, this study used videos as the augmented reality content. Subsequent studies could explore the role of interactive options or 3D visuals in children’s comprehension and retelling performance.

## Data Availability

The raw data supporting the conclusions of this article will be made available by the author, without undue reservation.
